# Identification of 3 key genes as novel diagnostic and therapeutic targets for OA and COVID-19

**DOI:** 10.3389/fimmu.2023.1167639

**Published:** 2023-05-22

**Authors:** Yiwei Zhang, Zhengwei Duan, Yonghao Guan, Tianyang Xu, Yuesong Fu, Guodong Li

**Affiliations:** Department of Orthopedics, Shanghai Tenth People’s Hospital, Tongji University School of Medicine, Shanghai, China

**Keywords:** osteoarthritis, SARS-CoV-2 infection, COVID-19, single-cell analysis, drug prediction

## Abstract

**Background:**

Corona Virus Disease 2019 (COVID-19) and Osteoarthritis (OA) are diseases that seriously affect the physical and mental health and life quality of patients, particularly elderly patients. However, the association between COVID-19 and osteoarthritis at the genetic level has not been investigated. This study is intended to analyze the pathogenesis shared by OA and COVID-19 and to identify drugs that could be used to treat SARS-CoV-2-infected OA patients.

**Methods:**

The four datasets of OA and COVID-19 (GSE114007, GSE55235, GSE147507, and GSE17111) used for the analysis in this paper were obtained from the GEO database. Common genes of OA and COVID-19 were identified through Weighted Gene Co-Expression Network Analysis (WGCNA) and differential gene expression analysis. The least absolute shrinkage and selection operator (LASSO) algorithm was used to screen key genes, which were analyzed for expression patterns by single-cell analysis. Finally, drug prediction and molecular docking were carried out using the Drug Signatures Database (DSigDB) and AutoDockTools

**Results:**

Firstly, WGCNA identified a total of 26 genes common between OA and COVID-19, and functional analysis of the common genes revealed the common pathological processes and molecular changes between OA and COVID-19 are mainly related to immune dysfunction. In addition, we screened 3 key genes, DDIT3, MAFF, and PNRC1, and uncovered that key genes are possibly involved in the pathogenesis of OA and COVID-19 through high expression in neutrophils. Finally, we established a regulatory network of common genes between OA and COVID-19, and the free energy of binding estimation was used to identify suitable medicines for the treatment of OA patients infected with SARS-CoV-2.

**Conclusion:**

In the present study, we succeeded in identifying 3 key genes, DDIT3, MAFF, and PNRC1, which are possibly involved in the development of both OA and COVID-19 and have high diagnostic value for OA and COVID-19. In addition, niclosamide, ciclopirox, and ticlopidine were found to be potentially useful for the treatment of OA patients infected with SARS-CoV-2.

## Introduction

Osteoarthritis (OA), the most prevalent joint illness, is a degenerative condition marked by joint inflammation and the loss of cartilage (1). OA mainly impacts load-bearing joints, such as the knee and hip, causing chronic pain and dysfunction in the affected joints, and thus seriously affecting the patient’s quality of life (2). Although the pathogenetic mechanisms of OA are diverse, previous studies have shown that inflammatory responses and joint immune disorders are significantly involved in the formation and development of osteoarthritis and affect the clinical presentation and prognosis of patients to varying degrees (3). In recent years, as the research on OA has gradually intensified, the relationship between OA and other diseases has gradually attracted the attention of researchers (4).

Corona Virus Disease 2019 (COVID-19) refers to severe acute respiratory syndrome coronavirus 2 (SARS-CoV-2) infection causing pneumonia, first discovered in 2019 (5). The COVID-19 pandemic poses serious challenges to the global economy and development (6). As research on COVID-19 progresses, increasing evidence suggests that COVID-19 is closely associated with immune dysregulation and abnormal inflammatory responses (7, 8). For example, Schimke et al. found that patients with severe COVID-19 were characterized by excessive neutrophil activation and that the degree of activation correlated with the severity of COVID-19 patients (9). Therefore, the relationship between COVID-19 and inflammatory disease and immune-related diseases has been extensively studied, for example, Hu et al. uncovered the relationship between COVID-19 and RA and identified genes that are significantly different and play an important role in both diseases (10). However, the shared molecular pathways and therapeutic strategies between OA and COVID-19 are rarely mentioned.

In a predictive model examining the survival of COVID-19 patients, we found OA to be a predictor associated with this model, which drew our attention (11). Interestingly, Paxlovid, a drug widely used to treat COVID-19, has been shown to accelerate cartilage degeneration in patients, thereby triggering or exacerbating osteoarthritis (12). In addition, Lauwers et al. found in their study that both COVID-19 and OA patients had dysfunction of the renin-angiotensin system and inflammatory response (13). Based on these findings, the potential association between COVID-19 and OA and its molecular mechanisms urgently needs to be further explored to find effective therapeutic plans or drugs to deal with OA caused or exacerbated by SARS-CoV-2 infection. For this purpose, in this study, we used RNA-sequencing (RNA-seq) data from OA and COVID-19 patients for bioinformatics, machine learning algorithms, and single-cell analysis to explore shared molecular pathways and therapeutic strategies between OA and COVID-19 for the first time. Finally, we identified key genes that contribute to the development of both OA and COVID-19 and then used network pharmacology to filter out potentially effective therapeutic agents for the OA patients infected with SARS-CoV-2.

## Materials and methods

### Data collection

The RNA-seq data of OA and COVID-19 used for the analysis in this paper were obtained from the Gene Expression Omnibus (GEO) database, including two datasets GSE114007 and GSE55235 of OA, and two datasets GSE147507 and GSE171110 of COVID-19. Since the aim of this study was to investigate the common pathogenesis between OA and COVID-19 at the genetic level, we excluded transcriptome sequencing data from 10 patients with rheumatoid arthritis from the OA validation dataset GSE55235. Similarly, we eliminated the high-throughput sequencing data of ferrets from the COVID-19 training dataset for this project because the species under investigation in this study is Homo sapiens. The details of the datasets are provided in **Table 1** and **Supplementary Table 1**.

**Table 1:** Information of datasets.

Disease	Dataset ID	Platform	Samples (control/disease)	Attribute
Osteoarthritis	GSE114007	GPL18573	18/20	Train set
Osteoarthritis	GSE55235	GPL96	10/10	Validation set
COVID-19	GSE147507	GPL18573	55/23	Train set
COVID-19	GSE171110	GPL16791	10/44	Validation set

### Analysis of gene differential expression

The “limma” (version 3.40.6) R package was used to filter differentially expressed genes (DEGs) between OA and COVID-19 patients and normal subjects, respectively, with log2 |FC| > 1 and FDR < 0.05 as screening criteria. Visualization of heat plots and volcano maps of DEGs between OA and normal in GSE114007 and COVID-19 and normal in GSE147507 implemented by the R packages “ggplot2” (version 3.3.6) and “pheatmap” (version 1.0.12).

### Weight gene co-expression network analysis

The R package “WGCNA” (version 1.71) was used for the implementation of Weight Gene Co-Expression Network Analysis (WGCNA) to capture the gene modules most associated with OA or COVID-19. Using the default parameters of the R package and based on weighted Pearson correlation, hierarchical clustering analysis is performed. Then, 4 and 11 were selected as the soft threshold power for GSE114007 and GSE147507 based on scale independence greater than 0.80 to assure physiologically significant scale-free networks. The adjacency matrix is constructed using weighted correlation values between genes and genes and transformed into a topological intersection lattice (TOM) to reduce noise and false correlation, which quantifies the tissue availability of a gene characterized by the fact that the correlation of any two genes is not nearly determined by their correlation but also depends on the interactions of other genes with which the two genes are correlated. The number of module genes was set to 50 and 0.25 was set as the cut threshold, and different gene modules were obtained according to the set criteria cut clustering results, indicated by branches of the clustering tree and different colors. Based on the calculated gene significance and module membership, the top three gene modules most associated with OA or COVID-19 were selected. Finally, 26 common genes between OA and COVID-19 were found using the Venn diagram.

### Functional analysis

Enricher (https://maayanlab.cloud/Enrichr/), a web-based tool for comprehensive analysis, was utilized to analyze the function and pathways of common genes between OA and COVID-19. In particular, pathway enrichment analysis included KEGG 2021 Human, Reactome 2022, and WikiPathway 2021 Human. Histogram of enrichment analysis results generated directly from the website.

### Key gene capture and validation

The least absolute shrinkage and selection operator (LASSO) logistic regression analysis was used to further screen out key genes from the 26 common genes. GSE114007of OA and GSE147507 of COVID-19 were analyzed using the LASSO algorithm respectively, and the screened genes were intersected to finally obtain 4 key genes. The differential expression analysis and Receiver Operating Characteristic (ROC) curves for key genes were implemented in the training and validation datasets using the “limma” (version 3.40.6) and “pROC” (version 1.17.0.1) R packages, with P > 0.05 considered statistically different.

### Immunity analysis of key genes

The CIBERSORT algorithm, implemented by the R package “CIBERSORT” (version 1.03), was used to analyze the immune cell composition of normal individuals, OA, and COVID-19 patients in GSE114007 and GSE147507 (14). The correlation of key gene expression levels with 22 immune cells in OA and COVID-19 patients was analyzed by Pearson correlation analysis. In addition, the expression levels of key genes in immune cells were demonstrated by the Human Protein Atlas (HPA) database (https://www.proteinatlas.org/) (15).

### Single-cell analysis of key genes

Single-cell analysis using the Deeply Integrated human Single-Cell Omics (DISCO) database (https://www.immunesinglecell.org/) that contains whole blood scRNA-seq data from 717 COVID-19 patients was performed to explore the expression patterns of key genes (16).

### The regulation network of common genes

The regulatory networks of common genes and transcription factors (TFs) were built, using the JASPAR database on the Networkanalyst platform, to identify the major variants common to OA and COVID-19 at the transcriptional level and to gain more insight into the regulatory mechanisms of TFs on common genes. Similarly, the MiRTarBase database was used to structure the regulatory network of common genes and miRNAs to investigate the post-transcriptional modifications of common genes by miRNAs.

### Drug prediction and molecular docking

Through the Enrichr website, drug predictions were performed using the Drug Signatures Database (DSigDB) for the common gene between OA and COVID-19 trying to discover drugs that could be used to treat SARS-CoV-2-infected OA patients. The specific filtering method is as follows. First, we enter 26 common genes between OA and COVID-19 into the website and click “submit”. After that, we select the DSigDB database in “Diseases/Drug” on the pop-up screen to get the drugs and details acting on the common genes. In addition, 3D structures of small molecule drugs for molecular docking were downloaded from the PubChem (https://pubchem.ncbi.nlm.nih.gov/) database, and structures of proteins encoding key genes were obtained from the UniProt (https://www.uniprot.org/) database. The AutoDockTools (version 1.5.7) was performed for docking experiments and free energy of binding calculations, and the final results were visualized using PyMOL software.

## Results

### Analysis of gene differential expression for OA and COVID-19

**Figure 1:**
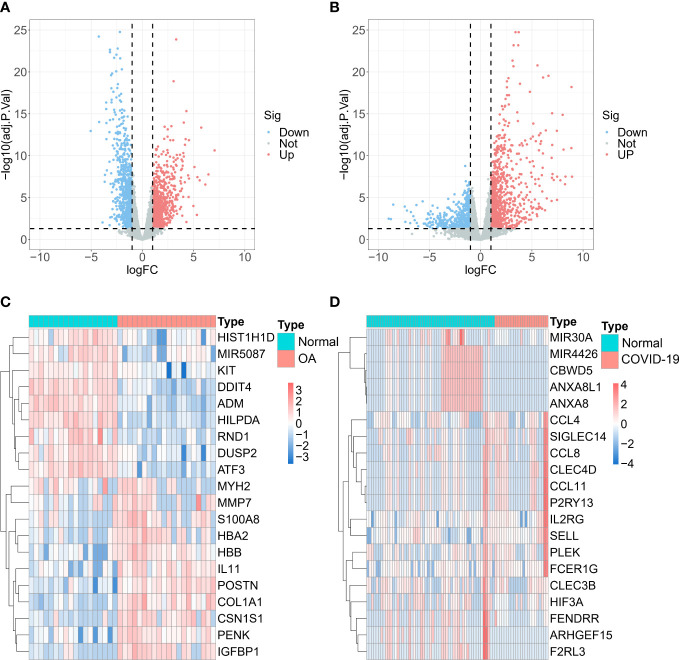
Differentially expressed genes (DEGs) analysis. Volcano maps and heatmaps of **(A, C)** GSE114007 and **(B, D)** GSE147507.

To obtain DEGs related to OA, we analyzed the analysis of gene differential expression with GSE114007. Using log2 |FC| > 1 and FDR < 0.05 as criteria, we screened for 991 up-regulated and 915 down-regulated DEGs related to OA (**Figures 1A, C**). Likewise, the analysis results in GSE147507 showed that 984 up-regulated and 542 down-regulated DEGs were found in normal and COVID-19 patients (**Figures 1B, D**).

### WGCNA and common genes acquisition

**Figure 2:**
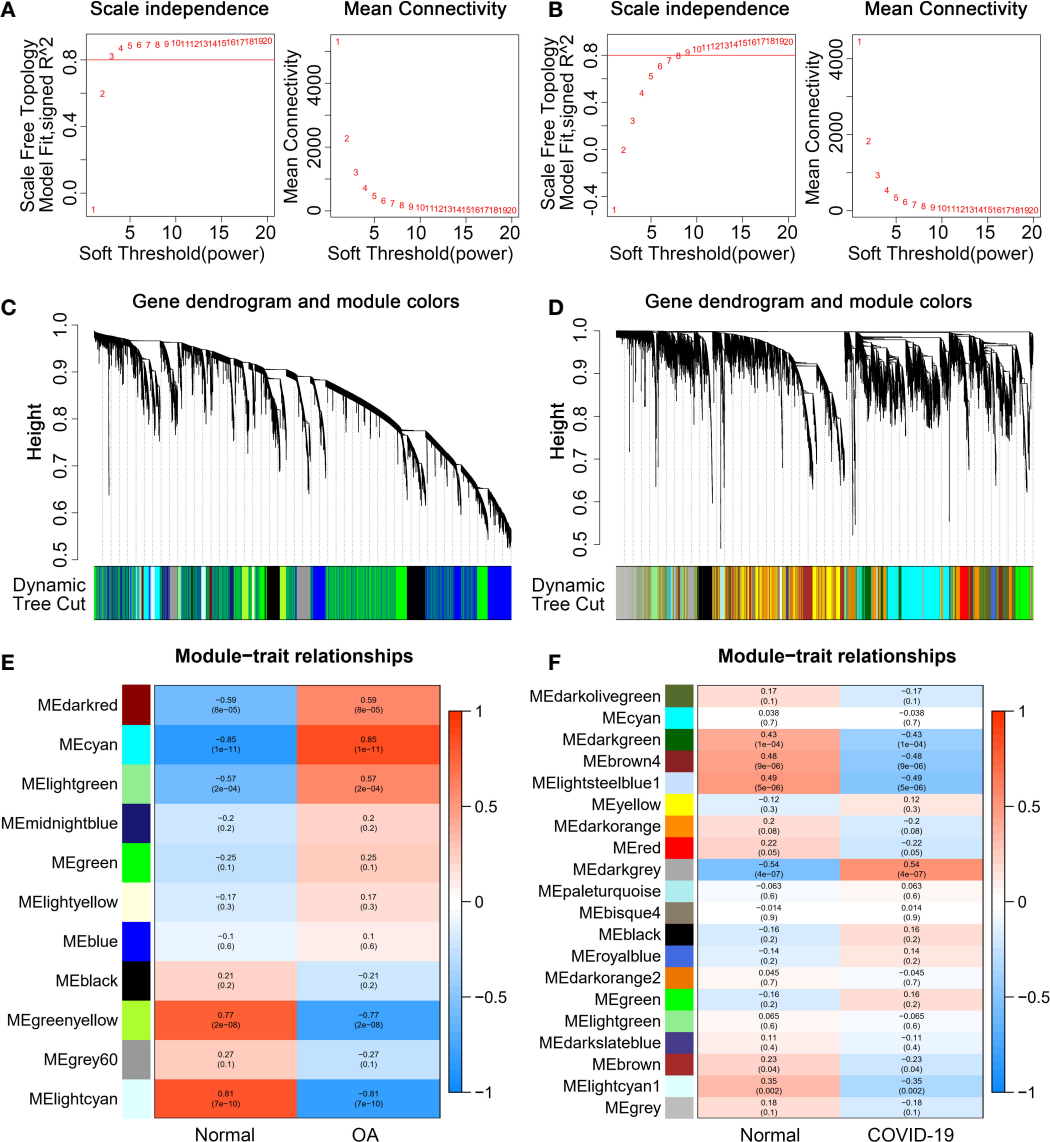
Weight Gene Co-Expression Network Analysis (WGCNA). **(A, B)** Independence and mean connectivity analyses to determine the soft threshold for scale-free networks of GSE114007 and GSE147507. **(C, D)** The Gene dendrograms of GSE114007 and GSE147507. The tree diagram’s colorful rows beneath it showed that modules were assigned depending on dynamic tree cutting. **(E, F)** The correlations between modules and traits of GSE114007 and GSE147507.

**Figure 3:**
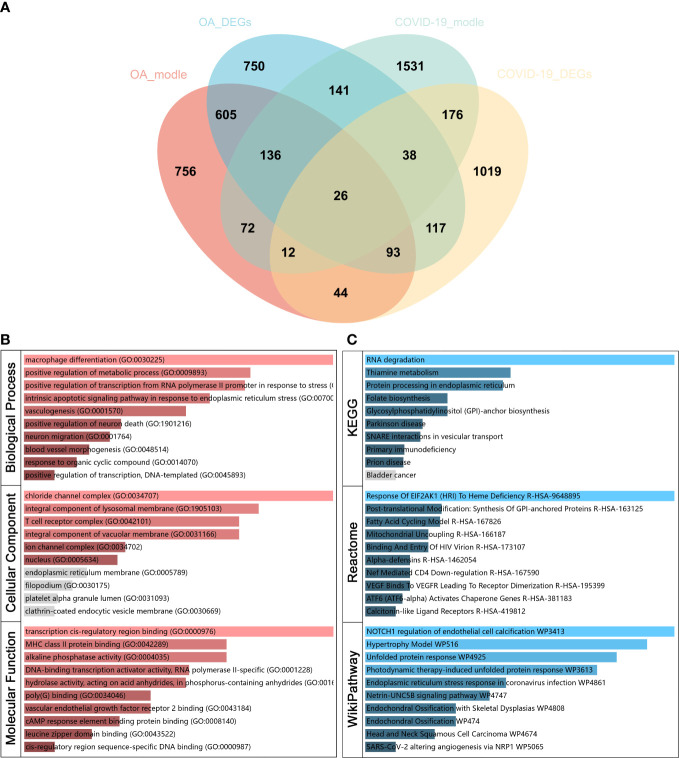
Identify common genes related to osteoarthritis (OA) and Severe coronavirus disease 2019 (COVID-19) and functional analysis. **(A)** Venn diagram of 26 common genes related to OA and COVID-19. **(B)** Gene Ontology (GO) and **(C)** pathway enrichment analyses of common genes.

The WGCNA was performed to identify the set of genes that vary highly synergistically with COVID-19 and OA. To make the co-expression network more consistent with the characteristics of the scale-free network, we drew the scatter plot of the fit index versus power value and the scatter plot of the average connectivity versus power value, respectively. With scale independence > 0.80, 4 and 11 are chosen as the soft threshold for GSE114007 and GSE147507 (**Figures 2A, B**). Then, based on weighted correlations, we carried out a hierarchical clustering analysis to obtain different gene modules, which were represented by branches of the gene dendrograms and different colors (**Figures 2C, D**). Besides, according to the calculated GS and MM, we plotted the heat map of gene modules and traits, which displayed that the MEcyan, MEgreenyellow, and MElightcyan modules were closely related to OA and the MEdarkgrey, MEbrown4, and MElightsteelblue1 modules were highly correlated with COVID-19 (**Figures 2E, F**). Finally, we identified 26 common genes between OA and COVID-19 by the veen diagram (**Figure 3A**).

### Functional analysis of common genes

To investigate the function of the common genes between OA and COVID-19 in disease development, GO and pathway enrichment analysis was performed. The GO analysis showed that the common genes were most significantly enriched in macrophage differentiation in BP, chloride channel complex in CC, and transcription cis-regulatory region binding in MF (**Figure 3B**). For pathway analysis, the common genes were most related to RNA degradation in KEGG, the response of EIF2AK1 (HRI) to heme deficiency in Reactome, and NOTCH1 regulation of endothelial cell calcification in WikiPathway (**Figure 3C**).

### LASSO logistic regression analysis and key genes identification

**Figure 4:**
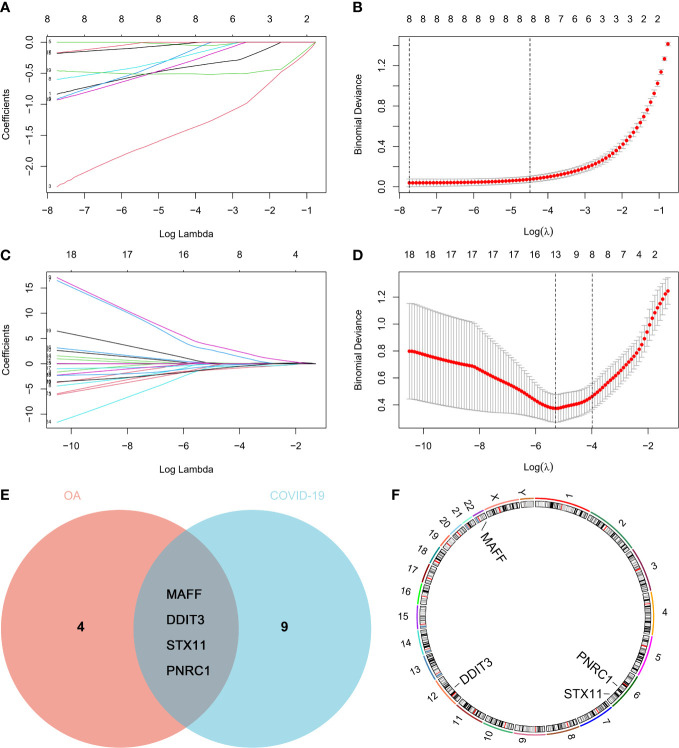
Key genes screened by the machine learning algorithm. The least absolute shrinkage and selection operator (LASSO) logistic regression analysis of 26 common genes in **(A, B)** GSE114007 and **(C, D)** GSE147507. **(E)** Venn diagram and **(F)** chromosome distribution of key genes.

Using LASSO logistic regression analysis, we attempted to screen for the genes with the greatest effect on OA and COVID-19 occurrence among the common genes and finally obtained 8 genes for OA and 13 genes for COVID-19 (**Figures 4A–D**). As shown in the veen diagram, the LASSO analysis results of OA and COVID-19 were crossed and four key genes were obtained(**Figure 4E**). Besides, the chromosome distribution of key genes was marked (**Figure 4F**).

### Key genes differential expression, ROC analysis, and validation

**Figure 5:**
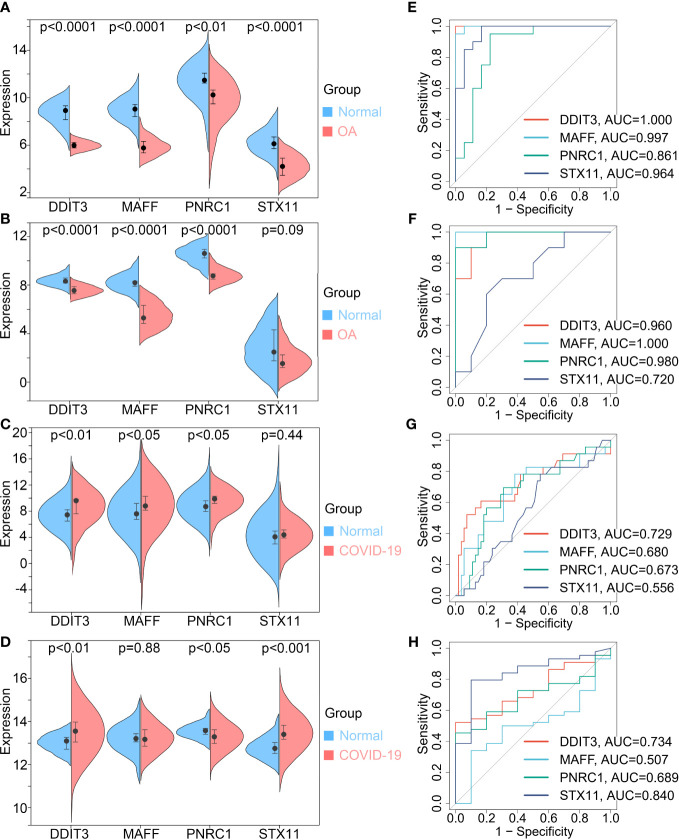
Key genes expression differential analysis and Receiver Operating Characteristic (ROC) curves. OA **(A, E)** train set GSE114007 and **(B, F)** validation set GSE55235. COVID-19 **(C, G)** train set GSE147507 and **(D, H)** validation set GSE171110.

The expression differential analysis was performed to compare the expression degrees of key genes in normal and disease. The results showed that the expression of key genes was reduced in OA patients and increased in COVID-19 patients compared with normal (**Figures 5A, C**). Then, we validated the results using the validation sets GSE55235 for OA and GSE171110 for COVID-19 (**Figures 5B, D**). However, we found that the differential expression of STX11 was not statistically significant in GSE55235 and GSE147507. In addition, Receiver Operating Characteristic (ROC) curves were plotted to detect the diagnostic performance of key genes for OA and COVID-19 and validated using GSE55235 and GSE147507 (**Figures 5E–H**). Collectively, these results suggest that DDIT3, MAFF, and PNRC1 play important roles in OA and COVID-19 pathogenesis, while STX11 lacks specificity. Therefore, we conducted further studies on DDIT3, MAFF, and PNRC1.

### Immunity analysis of key genes

**Figure 6:**
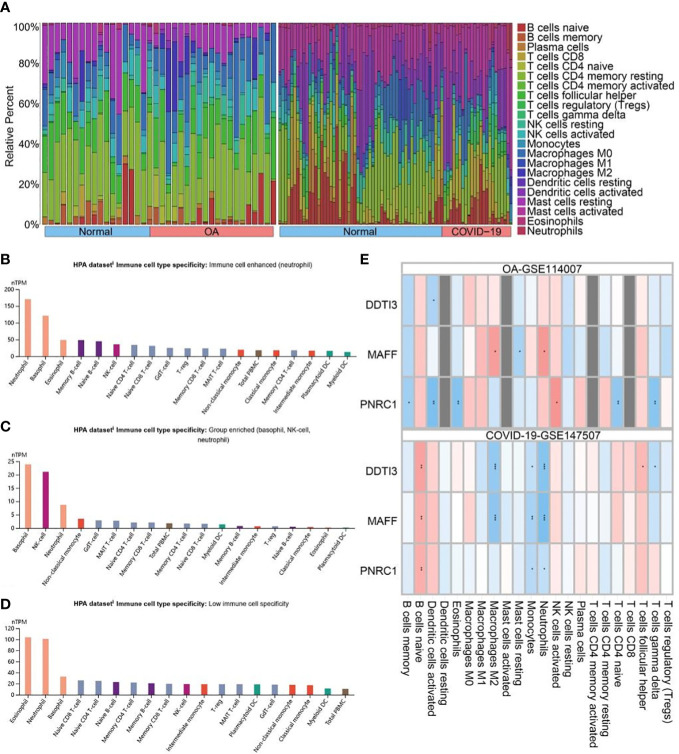
Immune analysis. **(A)** Immune cells overview diagram of GSE114007 and GSE147507. Histogram of **(B)** DDIT3, **(C)** MAFF, and **(D)** PNRC1 expression in 18 immune cells getting from the Human Protein Atlas (HPA) database. **(E)** Correlation analysis of the expression of key genes and immune cell infiltration in OA and COVID-19. Positive and negative correlations are denoted by the colors red and blue, respectively. *, p < 0.05; **, p < 0.01; ***, p < 0.001.

First, the histogram shows the immune cell composition profile of all samples (**Figure 6A**). To investigate the influence of key genes differentially expressed on the immune system, we analyzed the expression of key genes in immune cells using the HPA database (**Figures 6B–D**). The results showed that DDIT3 was highly expressed in neutrophils, MAFF was highly expressed in basophils, NK cells, and neutrophils, but PNRC1 was not specifically expressed. In addition, we investigated the correlation between the expression of key genes and the level of 22 immune cell infiltration in OA and COVID-19 patients (**Figure 6E**). We found that DDIT3 expression was significantly and negatively correlated with the level of dendritic cells activated infiltration, MAFF expression was significantly and positively correlated with the level of neutrophil and macrophages M2 infiltrations, and PNRCI was significantly and positively correlated with the level of NK cells activated infiltration and negatively correlated with the level of B cells memory, dendritic cells activated, eosinophils, T cells CD4 naïve, and T cells gamma delta infiltrations in OA patients, whereas DDIT3 was significantly and positively correlated with the level of T cells follicular helper and B cells naïve infiltrations but negatively correlated with T cells gamma delta, monocytes, neutrophils, and macrophages M2 infiltrations, MAFF was significantly and positively correlated with the level of B cells naïve infiltration but negatively correlated with monocytes, neutrophils, and macrophages M2 infiltrations, and PNRC1 expression was positively correlated with the level of B cells naïve infiltration but negatively correlated with monocytes and neutrophils infiltrations in COVID-19 patients.

### Single-cell analysis of key genes

**Figure 7:**
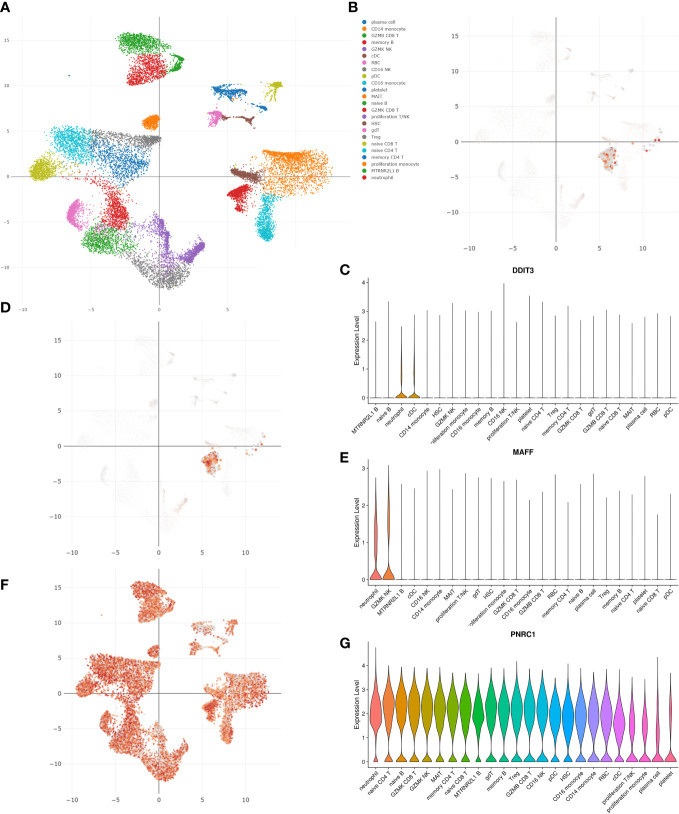
Single-cell analysis using the DISCO database revealed the expression patterns of three key genes in COVID-19 patients. **(A)** The UMAP plot of all blood cells in COVID-19 patients. The UMAP and violin plots of **(B, C)** DDIT3, **(D, E)** MAFF, and **(F, G)** PNRC1 expression in different cell clusters.

Utilizing blood single-cell RNA sequencing (scRNA-seq) data of 717 COVID-19 patients from the DISCO database, we analyzed the expression patterns of 3 key genes in COVID-19 patients (**Figure 7A**). As shown in the UMAP and violin plots, in COVID-19 patients, DDIT3 was highly expressed in neutrophils and cDC cells, MAFF was highly expressed in neutrophils and GZMK NK cells, and PNRC1 was highly expressed in almost all immune cells and blood cells (**Figures 7B–G**).

### The regulation network construction

**Figure 8:**
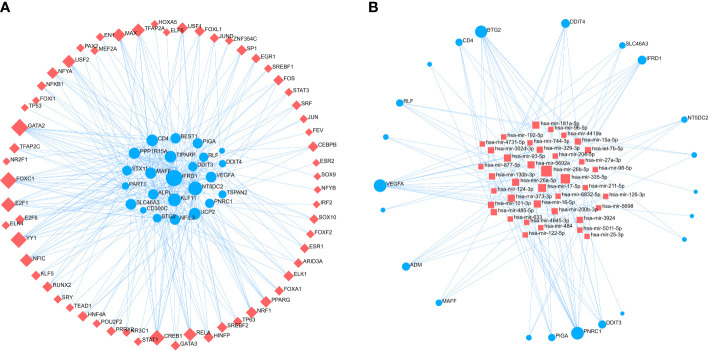
The regulatory networks of **(A)** common genes - TF and **(B)** common genes - miRNAs. Red square nodes are on behalf of TF and miRNAs, while common genes are denoted by the blue circle nodes. TF, Transcription Factors.

To identify major variants at the transcriptional level and get more insight into posttranscriptional regulatory mechanisms of common genes between OA and COVID-19, we constructed the regulatory networks of common genes - TF and common genes – miRNAs (**Figures 8A, B**). The results showed that the regulatory networks contain 62 TFs and 43 miRNAs. Taken together, the selection of therapeutic drugs for the common genes is aided by the understanding of posttranscriptional regulatory mechanisms provided by regulatory networks.

### Drug prediction and calculation of drug binding energy to key genes

**Figure 9:**
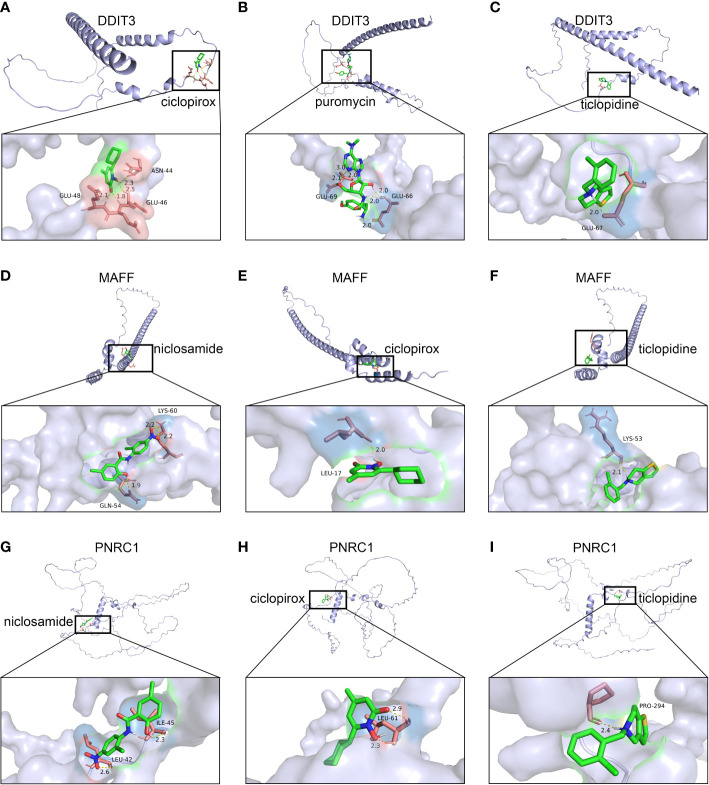
Molecular docking patterns for drugs and proteins corresponding to key genes. **(A)** ciclopirox, **(B)** puromycin, and **(C)** ticlopidine with DDIT3. **(D)** niclosamide, **(E)** ciclopirox, and **(F)** ticlopidine with MAFF. **(G)** niclosamide, **(H)** ciclopirox, and **(I)** ticlopidine with PNRC1.

Using the DSigDB database for drug prediction based on the common gene as a target for the treatment of OA and COVID-19, we selected the top 10 drugs by Adjusted P-value ranking, including niclosamide, ciclopirox, ticlopidine, puromycin, MG-132, fendiline, 15-delta prostaglandin J2, strophanthidin, mefloquine and prenylamine (**Table 2**). In addition, we used Autodock software to predict the free energy of binding and binding mode of niclosamide, ciclopirox, ticlopidine, puromycin, and MG-132 to key genes (**Table 3**). In addition, we selected the top three drugs with the strongest activity on DDIT3, MAFF, and PNRC1, and mapped the molecular binding patterns. (**Figures 9A–I**). The results indicated that niclosamide, ciclopirox, and ticlopidine showed high activity on all three key genes and therefore might be potential therapeutic agents for OA and COVID-19.

**Table 2:** Prediction of potential therapeutic agents.

Drug	Adjusted P-value	Molecular Formula	Structure
niclosamide	3.48E-13	C_13_H_8_Cl_2_N_2_O_4_	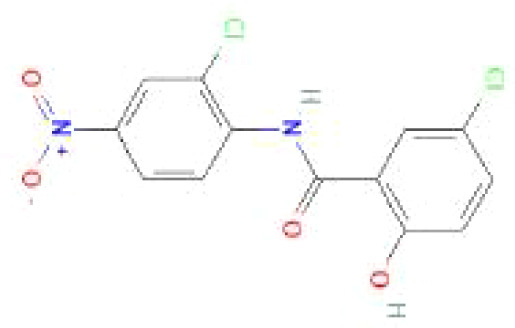
ciclopirox	7.15E-12	C_12_H_17_NO_2_	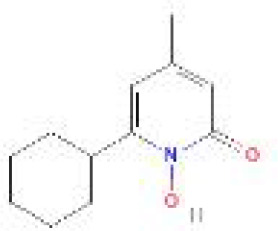
ticlopidine	8.35E-11	C_14_H_14_ClNS	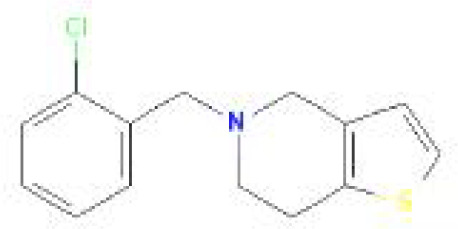
puromycin	8.35E-11	C_22_H_29_N_7_O_5_	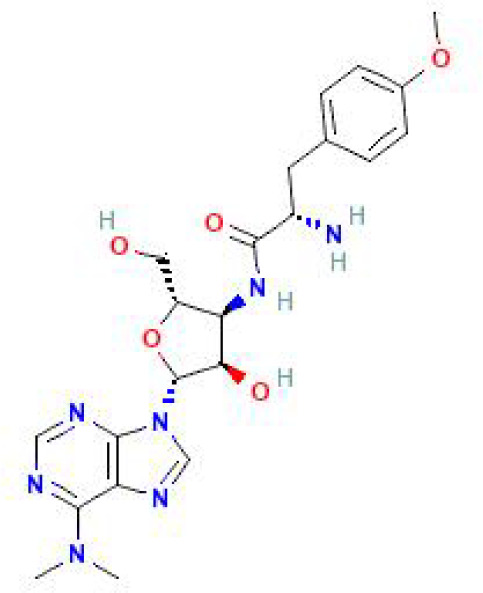
MG-132	2.00E-10	C_26_H_41_N_3_O_5_	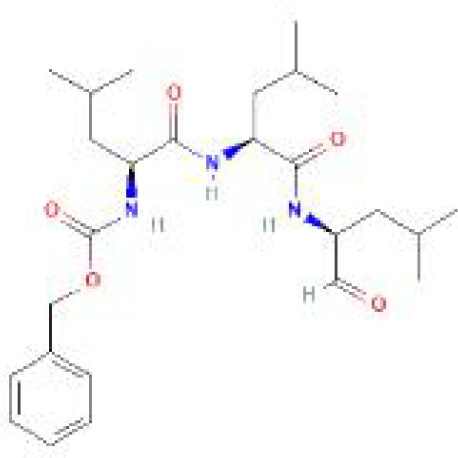
fendiline	3.42E-10	C_23_H_25_N	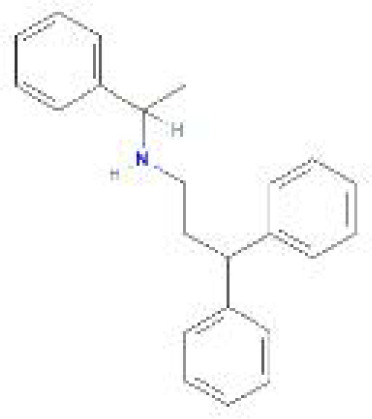
15-delta prostaglandin J2	4.13E-10	C_20_H_30_O_4_	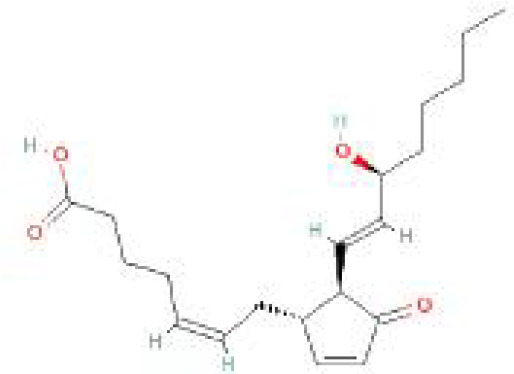
strophanthidin	7.81E-10	C_23_H_32_O_6_	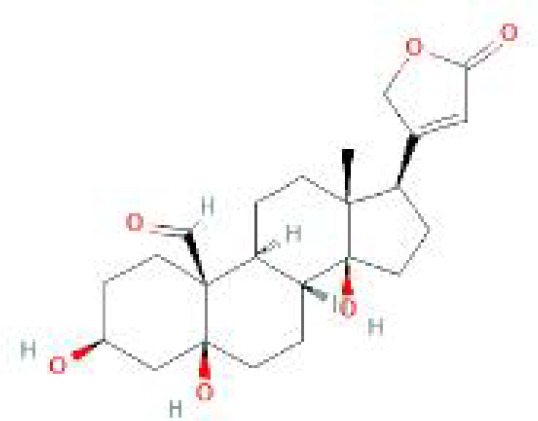
mefloquine	1.16E-09	C_17_H_16_F_6_N_2_O	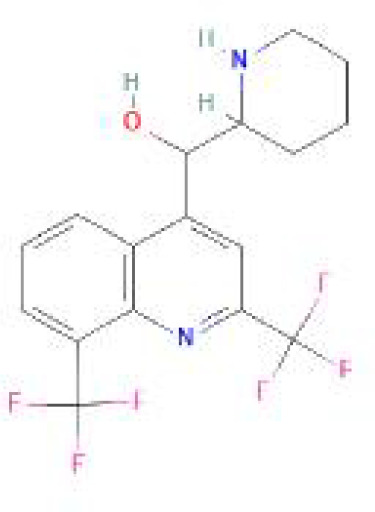
prenylamine	1.17E-09	C_24_H_27_N	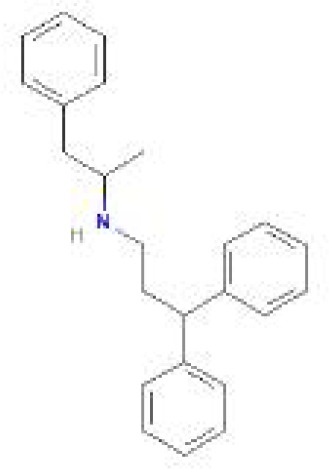

**Table 3:** The free energy of bindings between the top five drugs and the three key genes.

Drug	Free Energy of Binding (kcal/mol)		
	DDIT3	MAFF	PNRC1
niclosamide	-4.38	-4.62	-3.83
ciclopirox	-4.4	-4.26	-3.73
ticlopidine	-5.38	-4.16	-4.18
puromycin	-4.72	-1.62	-2.13
MG-132	-1.1	-0.5	-1.2

## Discussion

Since the onset of the COVID-19 outbreak, the cumulative number of confirmed cases worldwide has exceeded 700 million, of which over 6 million have died (https://covid19.who.int/). For the treatment of COVID-19 patients, several brand-new antiviral drugs have been created. However, these antiviral drugs can cause damage to the skeletal muscle system, such as cartilage degeneration (17). In addition, studies have shown that immune dysregulation is prevalent in patients with severe COVID-19 (18). Interestingly, cartilage degeneration and disturbances in the immune microenvironment are the main features of OA, which is the most common joint disease. Although researchers have noted a common clinical presentation and some potential associations between COVID-19 and OA patients, further studies on the shared molecular pathways and therapeutic strategies between these two diseases are needed (19).

In a predictive model examining the survival of COVID-19 patients, we found OA to be a predictor associated with this model, which drew our attention (11). Therefore, to investigate the potential link between COVID-19 and OA, we researched all the literature related to COVID-19 and OA and found that most studies focused on changes in treatment, diagnosis, and recovery status of OA patients during COVID-19 isolation (20, 21). However, our attention was drawn to the study by Veronesi et al. who hypothesized that COVID-19 infection causes a strong inflammatory response that leads to damage to the skeletal muscle system of patients (22). Unfortunately, to this date, no studies have reported a potential connection between OA and COVID-19 in terms of shared molecular changes and their mechanisms and therapeutic strategies. Therefore, in this paper, we explored for the first time the potential links and molecular mechanisms between OA and COVID-19 using bioinformatics, machine learning algorithms, and single-cell analysis. Firstly, the DEGs between OA and COVID-19 patients and normal subjects were identified separately. Then, we used WGCNA to identify genes in the modules most associated with OA and COVID-19 and crossed them with DEGs to find 26 common genes involved in OA and COVID-19 occurrence. Functional and pathway enrichment analysis is an important research method in bioinformatics, which helps to investigate the molecular mechanisms and regulatory roles of genes. Therefore, we performed functional and pathway enrichment analyses of 26 common genes, including GO enrichment analyses, KEGG pathway analyses, Reactome pathway analyses, and WikiPathway pathway analyses. The results indicate that the common genes of OA and COVID-19, in terms of GO, are mainly enriched in biological functions related to immunity, such as macrophage differentiation, T cell receptor complex, and MHC class II protein binding, and in KEGG pathway enrichment analysis are significantly enriched in pathways such as Primary immunodeficiency. These results are consistent with our finding that immune dysregulation is involved in both OA and COVID-19 patients. In addition, Mitochondrial Uncoupling in Reactome pathway analysis results in the involvement of OA onset and progression, as well as in the oxidative stress regulation and status of inflammation in COVID-19 patients (23, 24). Similarly, in WikiPathway pathway analysis results, SARS-CoV-2 altering angiogenesis *via* NRP1 and Endochondral Ossification are involved in the progression of COVID-19 and OA, respectively. Furthermore, a study showed that curcumin, which has anti-inflammatory and antioxidant effects, can be used to treat a variety of diseases, including COVID-19 and OA, which also suggests that inflammation and oxidation may be the shared pathogenic mechanisms in COVID-19 and OA (25). More importantly, in one study, Lauwers et al. found there were abnormal inflammatory responses and pain in COVID-19 patients, which is also characteristic of OA, due to endothelial dysfunction and adipose tissue dysfunction mediated by ACE2. Therefore, they hypothesized that dysfunction of the renin-angiotensin system and inflammatory response are common pathogenic molecular pathways in patients with COVID-19 and OA (13). Based on the above results, we are more certain that there are similar pathological processes and molecular changes between OA and COVID-19, which are mainly related to excessive inflammatory response. To further explore the molecular changes that play the most critical role in the formation and development of two diseases, we used the machine learning algorithm, LASSO logistic regression analysis, to screen for four key genes, DDIT3, MAFF, PNRC1, and STX11, out of 26 common genes. Differential gene expression is important for revealing the occurrence of disease at the genetic level, so we performed differential expression analysis of key genes in the train set GSE114007 and GSE147507 of OA and COVID-19, and verified the results in the validation sets GSE55235 for OA and GSE171110 for COVID-19. In a further step, we plotted ROC curves to analyze the predictive power of key genes for OA and COVID-19 in the train and validation sets. However, we found that the differential expression of STX11 was not statistically significant in GSE55235 and GSE147507. We, therefore, decided to abandon STX11 and carried out further research on DDIT3, MAFF, and PNRC1. Moreover, we noticed that DDIT3, MAFF, and PNRC1 were better predictors for OA, with AUC values greater than 0.85 in both the training and validation sets of OA, and may serve as new targets and perspectives for the diagnosis and treatment of OA. Therefore, we compared them with the diagnostic markers of OA identified in other published studies. In the study of Xia et al, ferroptosis-related genes such as CDKN1A and EGR1 were found to be potential diagnostic markers for OA, with the highest diagnostic value being JUN, which had AUC values as high as 0.989 and 0.96 in the ROC curves of the experimental and validation groups, but its diagnostic value was still lower than that of DDIT3 and MAFF, which we found to be the highest diagnostic markers for OA, with AUC values as high as 1.000, 0.997, 0.960, and 1.000 in the experimental and validation groups, respectively (26). In the study of Hu et al., we found the same result that DDIT3, MAFF, and PNRC1 had better diagnostic values (27). Although the diagnosis of COVID-19 is currently based on antigen testing and SARS-CoV-2 nucleic acid testing, the three key genes identified in our study are still important for the diagnosis of patients who suffer from both COVID-19 and OA. DNA damage-inducible transcription 3 (DDIT3), which increases apoptosis and is associated with adipogenesis and erythropoiesis, is significantly overexpressed in nasal epithelial cells with a high SARS-CoV-2 viral load and upregulates the TNF- a pathway (28). However, the pathogenic mechanism of DDIT3 in OA has not been elucidated, which may provide a new direction for future research on OA. MAFF, a key transcription factor associated with inflammation and lipid metabolism, has been shown to be significantly downregulated and play a critical role in OA (29). In addition, high MAFF expression can upregulate ACE2 and thus increase the inflammatory response in COVID-19 patients, resulting in poor prognosis (30). In contrast, the role of PNRC1, a marker with high diagnostic value for both OA and COVID-19, has not been investigated in OA and COVID-19, which provides a new perspective to study the potential connection and common pathogenesis between OA and COVID-19.

As we found in the functional analysis that immune disorders may be a shared pathological process and molecular changes between OA and COVID-19, we sought to explore the relationship between the expression of 3 key genes and pathological alterations in the immune microenvironment of OA and COVID-19 patients. To begin with, using the HPA database to explore the expression of three key genes in normal human blood immune cells, we discovered that DDIT3 was highly expressed in neutrophils, MAFF was highly expressed in basophils, NK cells, and neutrophils, but PNRC1 was not specifically expressed in immune cells. In addition, we investigated the correlation between the expression of key genes and the level of 22 immune cell infiltration in OA and COVID-19 patients with the CIBERSORT algorithm, which is shown in detail in the results. However, the CIBERSORT algorithm, which is mainly based on mRNA-seq data to analyze the immune cell composition of samples, applies to the analysis of immune cell composition in all diseases and normal subjects, which determines that its calculation results in different diseases will have a certain bias. Therefore we repeated this calculation 1000 times in the hope of minimizing bias. In addition, since it mainly calculates the immune cell composition of the sample and cannot express the gene expression of different immune cells in a given disease, we further performed single-cell analysis to investigate the expression patterns of DDIT3, MAFF, and PNRC1 in the immune cells of COVID-19 patients. Subsequently, to explore the specific role of the three key genes in the pathogenesis of COVID-19 development, we performed the single-cell analysis of scRNA-seq data from COVID-19 patients using the DISCO database. Comparing the results of the analysis with those of the HPA database, we found that the expression of DDIT3, MAFF, and PNRC1 in immune cells was the same as normal, indicating that the expression pattern of key genes in immune cells did not change during the progression of COVID-19. Increased neutrophils and decreased lymphocytes in the blood have been shown in many studies to be characteristic of severe COVID-19 patients and, more importantly, SARS-CoV-2 activates and induces the release of large numbers of neutrophil extracellular traps (NETs) from aggregated neutrophils, which can directly lead to lung epithelial cell damage and immune thrombosis, resulting in poor prognosis and increased risk of mortality (31, 32). In addition, Schimke et al. found that patients with severe COVID-19 were characterized by excessive neutrophil activation and that the degree of activation correlated with the severity of COVID-19 patients (9). Interestingly, the results of our study indicate that DDIT3, MAFF, and PNRC1 are all highly expressed in neutrophils, which strongly supports the importance of DDIT3, MAFF, and PNRC1 in the pathogenesis of COVID-19, and provides a new target for the treatment of severe COVID-19 patients with immune thrombosis caused by the substantial release of NETs. On the contrary, the role of macrophages and their transformation in the development of OA has been extensively studied, while the role of neutrophils has often been neglected. However, an increasing number of studies have shown that neutrophils are also involved in OA development and are required for OA progression (33, 34). Therefore, the key genes identified in this paper may provide new insights into the role of neutrophils in the pathogenesis of OA and may further contribute to the understanding of the pathogenesis of OA.

The regulatory networks of common genes - TF and common genes - miRNAs were built to identify the major variants common to OA and COVID-19 at the transcriptional level and to gain more insight into the regulation of common genes by transcription factors and post-transcriptional modification mechanisms of common genes by miRNAs. The networks showed that GATA2, FOXC1, CREB1, YY1, and TFAP2A were identified as the TFs most associated with common gene regulation, and hsa-mir-26b-5p, hsa-mir-26a-5p, hsa-mir-335-5p, hsa-mir-17-5p, and hsa-mir-98-5p were found to be the miRNAs with the highest correlation to common genes. Similarly, Huang et al. in a study identified YY1, CREB1, and GATA2 to be critical TFs involved in the regulation of COVID-19 patients at the genetic level in their pathogenesis (35). In addition, He et al. found that high expression of miR-204-5p downregulated FOXC1 expression in OA patients thereby suppressing the inflammatory response of synovial fibroblasts of OA (36). Paniri et al. discovered that hsa-mir-26b-5p might affect the susceptibility of COVID-19 patients to antiviral drugs (37). In addition, Rasheed et al. found that hsa-mir-26a-5p might be a new drug target for the treatment of OA due to its ability to modulate the NF-κB pathway in OA patients (38). In a study on key miRNAs for SARS-CoV-2 infection, hsa-mir-17-5p and hsa-mir-98-5p were found to play a key role in COVID-19 (39). The mechanism of action of these TFs and miRNAs in OA patients infected with SARS-CoV-2 has to be further studied, although several studies indicate they are important for the development and treatment of OA or COVID-19.

Since many antiviral drugs, such as Paxlovid, have been shown to accelerate cartilage degeneration in patients, thereby triggering or exacerbating OA, we tried to find suitable drugs for OA patients infected with SARS-CoV-2. Finally, we identified niclosamide, ciclopirox, and ticlopidine as potential therapeutic agents for the treatment of OA patients infected with SARS-CoV-2 by performing drug prediction on the shared genes of OA and COVID-19, and molecular docking and the free energy of binding estimation of predicted drugs with DDIT3, MAFF, and PNRC1 using Autodock software. Niclosamide, a widely used anthelmintic, has been shown in numerous studies to be a potential therapeutic agent for COVID-19. In a cellular experiment, Jeon et al. found that niclosamide had extremely high anti-SARS-CoV-2 efficacy (40). Brunaugh et al. similarly demonstrated the therapeutic efficacy of niclosamide on COVID-19 in a mouse model of COVID-19 (41). In addition, clinical trials conducted by Backer et al. and Cairns et al. have demonstrated the safety and efficacy of niclosamide for the treatment of SARS-CoV-2 patients (42, 43). Significantly, the treatment of patients with severe COVID-19 is still dominated by hormonal therapy such as dexamethasone, however, heavy hormone therapy can trigger secondary bacterial infections and immune dysregulation in patients, which is the main cause of death in COVID-19 patients. Whereas in studies for the treatment of COVID-19, niclosamide has shown not only the effects of inhibition on SARS-CoV-2 replication but also extremely strong bacteriostatic and immunomodulatory properties, which can greatly reduce the risk of death in patients with heavy COVID-19 (44–46). Although niclosamide has not been reported for the treatment of OA, we can speculate that niclosamide has an important value for the treatment of OA patients infected with SARS-CoV-2 based on its unique modulatory effect on the over-activation of the immune system, which is one of the mechanisms for the development of OA. Severe COVID-19 patients have a large number of lung microthrombi due to disruption of the immune system and severe destruction of alveolar endothelial cells, which can cause hypoxemia resulting from alveolar ventilation dysfunction and increase the risk of death in patients (47, 48). Moreover, immune system disorders can lead to a hypercoagulable state and arteriovenous thrombosis, which is responsible for the high rate of thrombotic complications in COVID-19 patients (49). Therefore, prophylactic use of anticoagulants in severe COVID-19 patients may improve patient survival (50). Interestingly, ticlopidine, a platelet inhibitor, not only can be used as a prophylactic anticoagulant in patients with severe COVID-19, but was also shown to counteract the intense endoplasmic reticulum stress caused by the SARS-CoV-2 virus infection, which leads to severe COVID-19, in a study by Tesei et al. and therefore has therapeutic value in patients with COVID-19 (51). Although the use of ticlopidine for OA treatment has not been reported, our study found that the free energy of binding between ticlopidine and the three shared key genes of OA and COVID-19 was extremely low, implying a stronger binding power, which may provide a new perspective for thromboprophylaxis and molecular therapy in OA patients infected with SARS-CoV-2. In terms of ciclopirox, as a broad-spectrum antifungal agent, studies for the use in the treatment of both OA and COVID-19 have not been reported. However, as we discussed before, severe COVID-19 patients with heavy hormone therapy can cause bacterial and fungal infections, and OA patients whose pain cannot be relieved by common medications are also commonly treated clinically with joint cavity injections of hormonal drugs to relieve patient pain, which can cause fungal septic arthritis (52). Therefore, future studies of Ciclopirox for the treatment of fungal infections secondary to COVID-19 or OA are interesting.

Notably, the present study focused only on the immunologically relevant role of key genes in the development of OA and COVID-19, but its role in other dimensions of OA and COVID-19 pathogenesis, as well as in SARS-CoV-2-infected OA patients, awaits further investigation.

## Conclusion

In this study, we identified 26 common genes between OA and COVID-19 by WGCNA and screened three key genes DDIT3, MAFF, and PNRC1 using machine learning algorithms. The ROC curves showed high diagnostic power of the 3 key genes for both diseases In addition, using the single cell analysis we uncovered that key genes are involved in the pathogenesis of OA and COVID-19 through high expression in neutrophils. Finally, we identified niclosamide, ciclopirox, and ticlopidine as potential therapeutic agents for the treatment of OA patients infected with SARS-CoV-2 by drug prediction and simulated docking patterns to calculate the free energy of binding between key genes and drugs.

## Data availability statement

The datasets presented in this study can be found in online repositories. The names of the repository/repositories and accession number(s) can be found in the article/**Supplementary Material**.

## Author contributions

YZ and ZD designed the study and performed the computational analysis. YG, TX, and YF collected the study data and wrote the manuscript draft. GL revised the draft and offered financial support for the project. All authors contributed to the article and approved the submitted version.
