# Supplementary material for: Identification of 3 key genes as novel diagnostic and therapeutic targets for OA and COVID-19
Source: Front Immunol. 2023 May 22;14:1167639. doi: 10.3389/fimmu.2023.1167639 (PMC10239847; doi:10.3389/fimmu.2023.1167639)
Supplement: Supplementary file 1 [file Table_1.docx]

Supplementary Table 1 Clinical characteristics of samples in GSE114007

| **Characteristic** | **Control**  **n =18** | | | **OA**  **n =20** | **Total**  **n =38** |
| --- | --- | --- | --- | --- | --- |
| Age | |  | |  |  |
| Mean ± SD | | | 36.61±13.46 | 66.20±7.35 | 52.18±18.31 |
| Median (min-max) | | | 34.00(18.00,61.00) | 67.50(51.00,82.00) | 57.00(18.00,82.00) |
| Sex |  | | |  |  |
| Male | 13(34.21%) | | | 8(21.05%) | 21(55.26%) |
| Female | 5(13.16%) | | | 12(31.58%) | 17(44.74%) |
| Grade |  | | |  |  |
| 4 | | | 0 | 20(52.63%) | 20(52.63%) |
| 1 | | | 18(47.37%) | 0 | 18(47.37%) |
